# Effect of Micro-Mold Cavity Dimension on Structure and Property of Polylactic Acid/Polycaprolactone Blend under Microinjection Molding Conditions

**DOI:** 10.3390/polym13060887

**Published:** 2021-03-13

**Authors:** Meng Wang, Weiwei Ding, Yeping Xie, Lifan Zhang, Yinghong Chen

**Affiliations:** The State Key Laboratory of Polymer Materials Engineering, Polymer Research Institute of Sichuan University, Chengdu 610065, China; 2018223090088@stu.scu.edu.cn (M.W.); 18428391006@163.com (W.D.); xieyeping2021@163.com (Y.X.); zhanglifan1998@163.com (L.Z.)

**Keywords:** polylactic acid, polycaprolactone, blend, microinjection molding, mechanical properties

## Abstract

Microinjection molding is a novel frontier polymer processing strategy different from conventional ones. In this paper, three different cavity-sizes of micro-mold tools were firstly fabricated, and the influences of micro-mold cavity dimension on the phase morphology structure, crystallization and orientation, and mechanical performance of the microinjection molded polylactic acid (PLA)/polycaprolactone (PCL) blend microparts were carefully investigated accordingly. The results show that the reduction of the cavity size would result in much higher shear stress field and cooling temperature gradient, which is advantageous to the fibrillation and orientation of PCL-dispersed phase. Consequently, with decreasing the micro-mold cavity dimension from length 26 mm to 15 mm, the interfacial compatibility is improved, significantly increasing number of PCL fibers with smaller diameter are in situ formed in PLA matrix and their orientation degree also obviously increases, which is verified by SEM and 2D-WAXD measurements. The Differential Scanning Calorimetry (DSC) analysis shows that the decrease in cavity dimension causes the enhancement of PLA crystallization property due to shear-induced crystallization, which is reflected by the decreasing PLA cold crystallization temperature and increasing PLA crystallinity (almost doubling that of conventional macropart). As a result, the dynamic/static mechanical property measurements exhibit that with decreasing the cavity size, the storage modulus, and the loss modulus of PLA/PCL blend micropart increase, and the corresponding tensile strength, elongation at break, and Young’s modulus also present an obviously increasing tendency. The related investigations would provide some new spaces and insights for realization of high-performance of PLA/PCL blend micropart.

## 1. Introduction

With the rapid development of material science and biomedical technology (particularly the interventional treatment strategy), there is an obviously increasing demand for biomedical microdevices such as vascular clamps and bone screws. However, these biomedical microdevices are hardly fabricated by adopting the conventional polymer processing technologies such as compression molding, conventional injection molding, and so on. Fortunately, the newly emerging microinjection molding [1] has already been successfully applied in the preparation of microdevices including the biomedical one. As the frontier novel polymer processing technology, microinjection molding has been receiving more and more attention since its first emergence because it possesses various advantages such as good dimensional adaptability, low cost, high efficiency, and automatic mass process. Currently, microinjection molding has become one of the important branches in the micro-electromechanical systems (MEMS), which has the capability to accurately and quickly manufacture the miniaturized polymer parts [2]. Obviously, polymer materials are now still the optimal choice to manufacture the microdevices due to their characteristics of affordable price, easy processing, and easy functionalization [3,4]. However, the current polymer micro-devices are fabricated mostly from the single component of polymer and this is difficult to meet the actual request of micro-device for the multi-functionality and high performance [5]. Melt-compounding of two or more polymers or polymer and inorganic fillers could be an effective way to achieve the goal of functionality and high performance. Meanwhile, this is also an important way to prepare multifunctional and high-performance microdevices and represents one of the important directions for the development of microinjection molding technology.

As a biopolymer material, polylactic acid (PLA) possesses good biodegradability, biocompatibility, and good mechanical property (tensile strength and modulus) and is widely used in the fields of internal fracture fixation, tissue engineering scaffold and other biomedical products [6,7,8,9]. However, the small crystallinity, rigid skeleton structure [10], and tertiary carbon atom of PLA would lead to its obvious brittleness, poor heat resistance, and easy decomposition [11,12]. This hence greatly limits the wider application of PLA. In order to improve the toughness of PLA, many methods have been attempted by many researchers [6,7,8,9,10,11,12,13,14,15,16,17,18,19]. Polymer blending is one of the economical and effective methods to combine the advantages of the individual component polymers and hence improve the properties of the material [20,21,22]. One typical example is to melt-blend PLA with flexible polymer such as poly(ε-caprolactone) (PCL). PCL is a kind of biocompatible and biodegradable polyester with good flexibility and workability. Similarly, the insufficient stiffness and the higher cost hinder its wider application [13]. As mentioned before, blending PLA and PCL is a feasible strategy to combine their respective advantages. As a result, there are increasing studies carried out on PLA/PCL blend system in recent years [6,14,15,16,17,18,19]. However, as such the blend system was seldom used for microinjection molding investigations except for our previous work [23]. Obviously, PLA/PCL blend could be used as an ideal biomedical material candidate to fabricate the high-performance medical micro-devices such as micro bone screws and micro vessel clamps by adopting the microinjection molding technology. So, it would be interesting and of great significance to investigate the fundamental problems involved in microinjection molding of PLA/PCL blend.

Compared with the conventional injection molding, the microinjection molding generally adopts the much smaller cavity size. This accordingly makes that the microinjection molding is essentially different from the conventional injection molding [4,5]. On one hand, the shear rate of the former is far higher than that of the latter (the maximum shear rate of microinjection molding can be as high as ~10^6^ s^−1^, and comparatively the one of conventional injection molding can reach only ~10^3^ s^−1^); on the other hand, relative to conventional injection molding, the microinjection molding also exhibits the remarkably increased temperature gradient due to the substantial reduction in cavity size. The above changes would result in the significant difference in structure and property between micropart and micropart [23,24,25]. For example, for isotactic polypropylene system [24], the proportion of shear layer in micropart is much higher than that in the macropart. There are the highly oriented shish-kebab crystals formed in micropart but there are no such crystallization structures in macropart. In our previous work [23], it is found that PCL nanofibrils are formed in microinjection-molded micropart, but only PCL microfibrils are generated in conventionally injection molded macropart. This resulted in the much better mechanical property of the micropart than that of the macropart. In fact, even under the microinjection molding conditions, the different cavity and runner size would also cause the obvious difference in the shear stress and the temperature gradient of the corresponding different micro-cavity, which are surely reflected in the difference in structure and performance of microparts [26,27,28,29]. It is of great significance and great importance to investigate the influence of the cavity and runner size on the structure and performance of micropart. This would be also very important for understanding how to realize the high performance of micropart through designing an appropriate cavity size and structure. Accordingly, in this paper, we mainly investigated the influence of the cavity size on the shear rate distribution, crystallization, morphology, orientation, and mechanical performance of PLA/PCL blend under microinjection molding conditions. For this purpose, the micro tensile samples with three different cavity sizes, including 15 mm × 1 mm × 0.25 mm, 20 mm × 4 mm × 0.5 mm and 26 mm × 8 mm × 1 mm, were involved and the corresponding micro-mold tools were priorly fabricated. In order to highlight the influence of the mold-cavity size on the crystallization and orientation of PLA/PCL blend, the conventional injection-molded macropart with a much bigger size (150 mm × 17 mm × 4 mm) than that of the three types of microparts above mentioned was specifically introduced for a comparison. This will lay a good foundation for preparation of high-performance micro-devices of PLA/PCL blend to be applied in biomedical field in the future, including micro vascular clamp, micro bone screw, microneedle, and so on.

## 2. Experimental Section

### 2.1. Material

The 4032D poly (lactic acid) (PLA) with a weight-average molecular weight (M_w_) of about 100 kDa was bought from UNIC Technology Co., Ltd. (Suzhou, China). The PLA raw material we used has a melting flow index of 3.87 g/min (190 °C, 2.16 kg load) and a melting temperature of about 170 °C. The 600c poly(ε-caprolactone) (PCL) (M_w_ = 60 kDa) with a melting temperature of about 60 °C was supplied by Shenzhen Bright China Industrial Co. (Shenzhen, China).

### 2.2. Preparation of PLA/PCL Microparts

**Figure 1:**
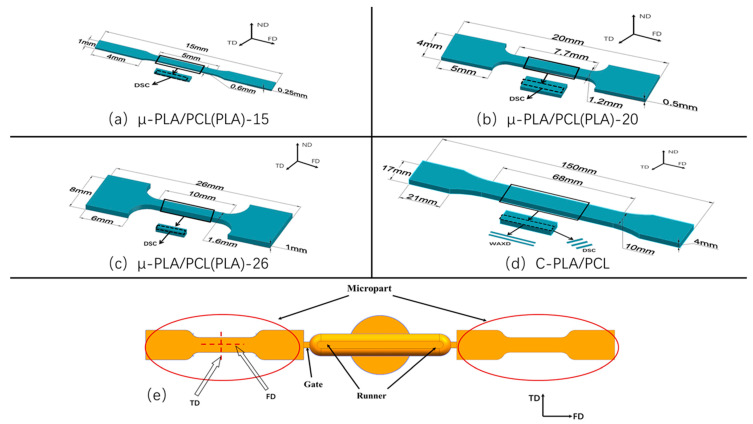
The dimension and shape of macropart and micropart with different cavity size: micropart 15 mm × 1 mm × 0.25 mm (μ-PLA/PCL-15 or μ-PLA-15) (**a**), 20 mm × 4 mm × 0.5 mm (μ-PLA/PCL-20 or μ-PLA-20) (**b**) and 26 mm × 8 mm × 1 mm (μ-PLA/PCL-26 or μ-PLA-26) (**c**); macropart 150 mm × 17 mm × 4 mm (C-PLA/PCL) (**d**); the configuration of micropart cavity with different size in micro-mold tool (**e**). The whole micropart is used for WAXD analysis. FD: flow direction, TD: transverse direction, ND: normal direction.

Before conducting the microinjection molding, we have designed and manufactured three cavity-sizes of micro-mold tools for preparation of PLA/PCL microparts. In order to avoid the influence of moisture on the hydrolysis of the raw materials during processing, the pellets of PLA and PCL polymers were dried in a vacuum oven at 40 °C for 24 h. The above dried PLA and PCL pellets (the weight fraction of PCL was fixed at 20%, i.e., the PLA/PCL weight ratio was fixed at 4/1) were well mixed in a high-speed mixer and the obtained mixtures were then extruded in a JSSJ-25/33 co-rotating twin-screw extruder (ϕ = 25 mm, L/D = 33 Chengguan Research Institute of Chemical Industry, Chengdu, China) with a rotation rate of 100 r/min in the range of 175–180 °C temperature. Then, the extrudates were cooled and pelletized into pellets. The dried pellets were microinjection-molded into microparts by using Battenfeld MicroPower 5 microinjection molding machine (Wittmann Battenfeld, Inc., Vienna, Austria). The used microinjection molding conditions are given below: injection speed of 400 mm/s, mold temperature of 40 °C, injection pressure of 1500 bar and melt temperature of 180 °C. For purpose of comparison, the dried pure PLA pellets were also microinjection-molded into microparts under the same conditions above mentioned. Figure 1a–e shows the dimension and shape of the microparts and macropart for tensile test used in this paper. For convenient discussion, the microinjection-molded pure PLA and PLA/PCL blend micropart samples with different size were marked as μ-PLA-x and μ-PLA/PCL-x, respectively, where μ means micro and x represents the length of the micro tensile sample; the conventionally injection-molded PLA/PCL blend macropart sample was marked as C-PLA/PCL, where C is the initial of “conventional” and means the conventional macropart.

## 3. Characterizations

### 3.1. Differential Scanning Calorimetry (DSC)

The Q20 (TA company, Newcastle, DE, USA) differential scanning calorimeter was applied to investigate the crystallization and melting behavior of PLA/PCL blend micropart. About 6–8 mg samples sealed in an aluminum crucible were firstly cooled from 40 °C to 0 °C at a cooling rate of 10 °C/min and then heated to 200 °C at a heating rate of 10 °C/min under nitrogen gas atmosphere with a flow rate of 50 ml/min. The PLA and PCL crystallinity degree in blend was calculated, respectively, using the formula shown below:

(1) $$X_{c,\;\mathrm{PLA}}\left(\%\right)=\frac{\Delta H_{m,PLA}-\Delta H_{c,PLA}}{\Delta H_{0,PLA}\omega_{PLA}}\times 100\%$$

(2) $$X_{c,\;\mathrm{PCL}}\left(\%\right)=\frac{\Delta H_{m,PCL}}{\Delta H_{0,PCL}\omega_{PCL}}\times 100\%$$

where $\omega_{PLA}$ and $\omega_{PCL}$ are the weight fraction of PLA and PCL in the blend, respectively; Δ*H_m,PLA_* and Δ*H_m,PCL_* are the melting enthalpy of PCL and PLA, respectively; Δ*H_c,PLA_* is the PLA cold crystallization enthalpy; Δ*H_0,PLA_* is the full PLA crystallization enthalpy (93.0 J/g) [30]; Δ*H_0,PCL_* is the full PCL crystallization enthalpy (139.3 J/g) [31].

### 3.2. Scanning Electronic Microscopy (SEM)

The micropart samples were fractured along the flow direction using liquid nitrogen. The fractured samples were etched by 1 wt% NaOH solution to get rid of part of PLA polymers. A thin gold layer was deposited on the fractured sections of all samples by vacuum spraying, and then the morphology of PCL-dispersed phase was observed on a Japan FEI INSPECT F scanning electron microscope at an accelerating voltage of 5 kV.

### 3.3. Two-Dimensional Wide-Angle X-ray Diffraction (2D-WAXD)

The 2D-WAXD measurements were performed on a BL16B1 beamline Synchrotron Radiation Facility (Shanghai, China) to investigate the sample crystallization and orientation behaviors. The wavelength of the X-ray was 0.124 nm and the diameter of the X-ray spot was 0.5 mm. The distance from sample to the detector was set at 130 mm. The micropart samples for test were shown in Figure 1. The X-ray was parallel to the normal direction (ND). The background of all 2D-WAXD data were extracted to carry out the qualitative comparison between different samples.

### 3.4. Mechanical Property Measurement

The tensile test of the micropart was carried out on an Instron 5567 tester (INSTRON company, Norwood, MA, USA) with a maximum loading of 1 kN and a cross-head speed of 1 mm/min.

### 3.5. Dynamical Mechanical Analysis (DMA)

In order to understand the thermomechanical properties and the interactions between materials at the molecular level, the dynamic mechanical analysis was performed toward the microinjection molded micropart. The DMA measurement was done on a DMAQ800 instrument (TA Instrument, Newcastle, DE, USA) in a multifrequency strain model. The apparatus was operated with a frequency 1 Hz in the range of −100–100 °C temperature at a heating rate of 3 °C/min.

## 4. Results and Discussion

### 4.1. Shear Rate and Temperature Gradient in Microinjection Molding

The polymer melt flow in micro-channels is significantly different from that in macro-channels. Obviously, the situation for the former is much more complex and the related molding theories for the traditional injection molding could not be applicable for microinjection molding. This is because the polymer melt processing is influenced by many factors such as the processing parameters and the mold cavity geometry and dimension. Wu et al. [32,33] found that compared with the processing parameters of mold temperature and injection speed, the mold cavity dimension and geometry have greater effect on the tensile strength of sample prepared by microinjection molding. The mold cavity dimension would cause a great change to the molding of micropart, which is mainly reflected in the following two aspects, i.e., the shear rate and the heat dissipation [34].

As proposed by Son et al. [35], when the ratio of width (w) to height (h) of the cross-section for the rectangular flow channel is smaller than 10 (w/h < 10), the rectangular die model can be applied to calculate the shear rate. Accordingly, the apparent shear rate of Newtonian fluid and the wall shear rate of non-Newtonian fluid in the slit die can be calculated by the following formula (3) and (4), respectively:

(3) $${\dot{\gamma }}_{a}=\left(\frac{6Q}{wh^{2}}\right)\left(1+\frac{h}{w}\right)f^{*}\left(\frac{h}{w}\right)$$

(4) $${\dot{(\gamma }}_{w}={\dot{\gamma }}_{a}\left(\frac{2}{3}\right)\left(\frac{b^{*}}{f^{*}}+\frac{a^{*}}{f^{*}}\frac{1}{n}\right)$$

where, ${\dot{\gamma }}_{a}$ and ${\dot{\gamma }}_{w}$ are the apparent shear rate and wall shear rate, respectively; *Q* is the volume flow rate; w and h are the width and the height of the flow channel, respectively; n is the power law index which is about 0.66 and the calculating process is not shown in this paper; a^*^, b^*^, and f^*^ are the corresponding geometric parameters. 

For the mold cavities with length of 15 mm, 20 mm, and 26 mm for microinjection molding, their w/h ratios are 0.6/0.25 = 2.4, 1.2/0.5 = 2.4, and 2/1 = 2, respectively, which are all smaller than 10. So, the rectangular model can be used to calculate the shear rate. The geometric characteristic parameters for calculation are provided in Table 1 [35].

**Figure 2:**
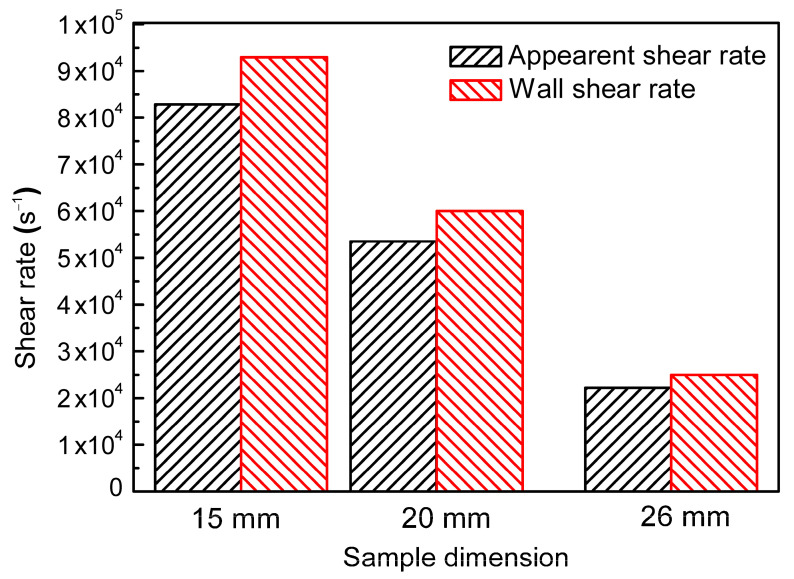
The shear rate in the mold cavity of micropart with different sample size.

Figure 2 shows the calculated shear rate in the mold cavity of micropart with different sample size. It can be seen that with decreasing mold cavity size to length 15 mm, the correlated wall shear rate increases to 9 × 10^4^ s^−1^. Obviously, the shear rate occurring in above mold cavity is an order of magnitude smaller than that occurring in the flow micro-channel which is about 10^6^ s^−1^ [35]. This is because the size of mold cavity in this study is much bigger than that of the flow micro-channel in literature. In the meantime, it is also noted that the shear rate of the polymer melt at the wall surface is greater than the apparent shear rate. As we know, the shear force field occurring during polymer processing and molding would exhibit a significant influence on the morphology, structure, and performance of the polymer material. As a result, such a high shear rate in microinjection mold cavity surely has a significant impact on the performance of the microinjection molded samples.

**Figure 3:**
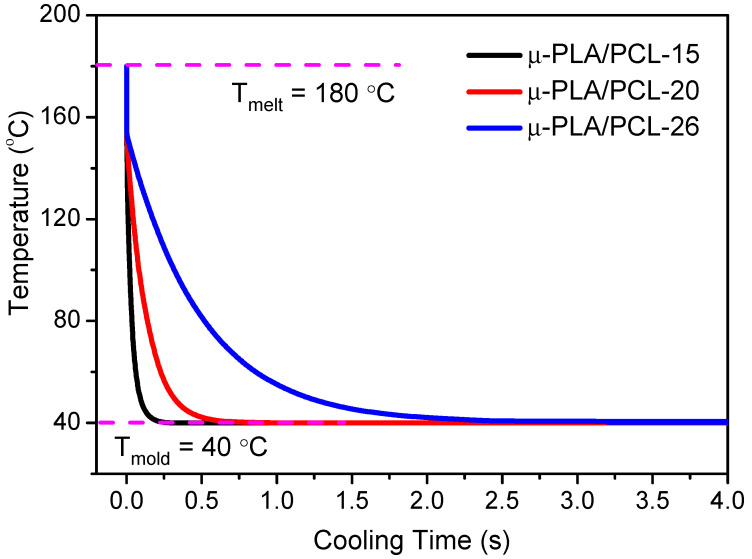
The cooling dissipation curves of microparts with different cavity size.

In addition, the heat dissipation in microinjection molding can be quantitatively described by the following equation [36]:

(5) $$T_{average}=T_{mold}+\frac{8}{\pi^{2}}\left(T_{melt}-T_{mold}\right)\exp\left(-\frac{a\pi^{2}t}{d^{2}}\right)$$

where, T_average_ is the real-time averaged temperature at cooling stage; *T_melt_* and *T_mold_* stand for polymer melting temperature and mold temperature, respectively; d is the thickness of the melt flow channel and *α* is the sample thermal conductivity (0.205 mm/s, and the PLA thermal conductivity can be approximately used as the one of all the samples). The corresponding heat dissipation curves and cooling parameters of different cavity size are shown in Figure 3 and Table 2, respectively. As can be seen from Figure 3, for μ-PLA/PCL-15 micropart with the smallest cavity size, the decrease to the temperature of mold cavity wall from melt temperature could finish within only 0.2 s.

Comparatively, for μ-PLA/PCL-26 micropart with the largest cavity size, it would take about 12 times longer time to reach the temperature of the mold cavity wall. What is more, the cooling rate also greatly increases from 58.3 K/s to 700 K/s as the mold cavity length decreases from 26 mm to 15 mm. In addition, the time to reach the temperature of mold cavity wall (T_mold_) reduces significantly as the cavity dimension decreases. How does the change of above two parameters caused by the different mold cavity dimension influence the formation of the microparts? To answer this question, the effects will be subsequently investigated in detail from the following aspects, including morphology, crystallization, orientation, mechanical property, etc.

### 4.2. Crystallization Melting Behavior

**Figure 4:**
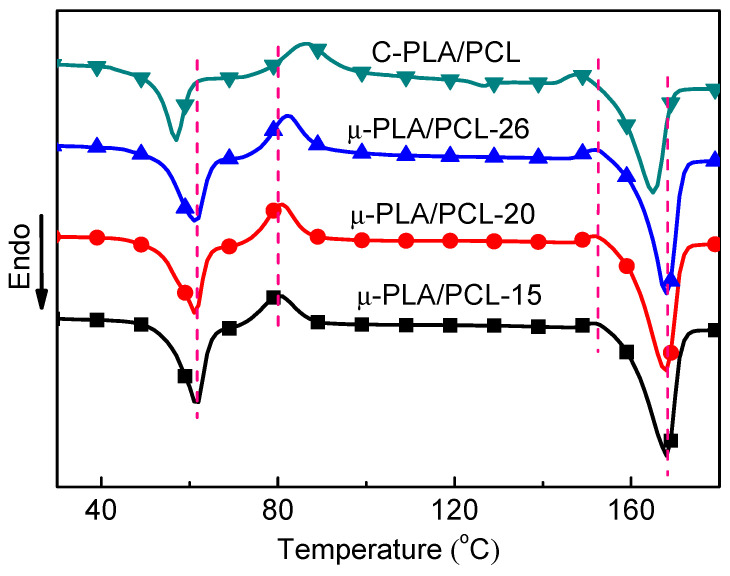
The Differential Scanning Calorimetry (DSC) heating curves of microparts with different cavity size and macropart.

The crystallization melting behaviors of different samples were investigated by using DSC analysis. The DSC results of microparts with different cavity size are shown in Figure 4 and Table 3. As a comparison, the DSC results of the conventionally injection-molded dumbbell-shaped sample (macropart) with dimension of length 150 mm, width 17 mm, and thickness 4 mm are also included. It can be seen that, in the DSC heating curves of PLA/PCL blend, four peaks can be identified: the overlapped PCL melting peak and PLA enthalpy relaxation peak (also the glass transition region) nearby 60 °C, the cold crystallization peak nearby 80 °C, the α-α′ crystallization phase transition peak nearby 150 °C and the PLA melting peak at about 165 °C. Compared with macropart, the cold crystallization temperature (T_cc_) and the cold crystallization melting enthalpy (ΔH_m_) of PLA in blend micropart with different cavity size significantly decrease. This reveals that compared with conventional injection molding, under microinjection molding conditions, the PLA macromolecular chains in blend are much more easily crystallized. Furthermore, it is also found that the melting temperatures (T_m_) of PLA and PCL in various microparts are higher than those in macropart. It is also worth noting that the crystallinity of PLA in blend micropart almost doubled that in blend macropart. In addition, compared with macropart, the crystallinity of PCL in micropart is also obviously increased. This indicates that the much stronger shear force field occurring in microinjection molding promotes the orientation of PLA macromolecular chains and further helps them be arranged into the crystal lattices, thus benefitting the crystallization of PLA, i.e., the shear induced crystallization. For μ-PLA/PCL blend microparts with different cavity size, with decreasing the cavity size, the cold crystallization temperature and cold crystallization melting enthalpy of PLA in blend also obviously decrease, indicating that the reduction in mold cavity size enhances the crystallization capability of PLA macromolecular chains in the corresponding micropart. It is also further found that, with decreasing the cavity size, the α-α′ crystallization phase transition peak gradually decreases in its intensity and even disappears finally. However, the melting temperature of PCL and PLA does not obviously change, but the crystallinity of PLA and PCL increases with cavity size decreasing (from 29.4% to 34.7% and 62.5% to 69.3%, respectively). This is because under the same microinjection molding conditions, with decreasing the cavity size, both the melt shear rate and the temperature gradient in mold cavity increase, which means that the shear stress field becomes increasingly strengthened and the melt solidification time becomes increasingly shortened. Under effect of the enhanced shear stress field, there are increasing numbers of macromolecular chains in molten state arrayed into the crystal lattices (more shear-induced crystallization) and they are solidified in a much shorter time than ever, leading to forming more crystals and hence the higher crystallinity of the polymer PLA and PCL. Above analyses and discussion indicate that the decrease in mold cavity size is beneficial for improvement of the crystallization performance of PLA. As is well known, the mechanical properties of the semi-crystalline polymers are closely related to the crystallization of their macromolecular chains. It can be predicted that the decrease in mold cavity size can be advantageous to enhancement of the mechanical properties of the microinjection-molded samples.

### 4.3. Phase Morphology Analysis

**Figure 5:**
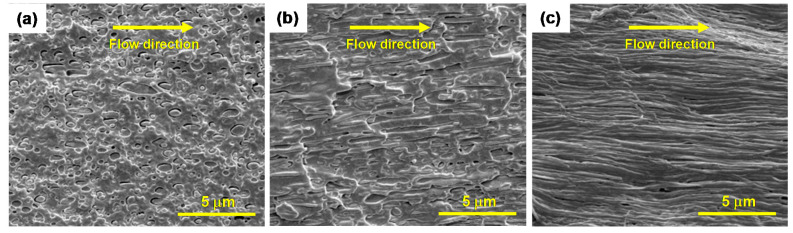
SEM photos of microparts with different cavity size along melt flow direction: (**a**) μ-PLA/PCL-26, (**b**) μ-PLA/PCL-20, and (**c**) μ-PLA/PCL-15.

Figure 5 shows the SEM micrographs of microparts of μ-PLA/PCL-2 μ-PLA/PCL-20 and μ-PLA/PCL-15 along melt flow direction. It can be seen that the PCL-dispersed phase of the μ-PLA/PCL-26 samples show the elongated ellipsoidal morphology and possesses a poor compatibility with PLA matrix due to the obvious interfacial gaps between both phases. With decreasing the cavity size, there are smaller and more numbers of PCL fiber-like structures in situ formed in the PLA matrix along the flow direction. Additionally, the length of PCL fibers increases and, however, their diameter continually decreases (some are even in the range of nanometers). In addition, the interfacial compatibility presents an increasingly enhancing tendency with decreasing the cavity size. The reasons for the changes of the PCL-dispersed phase morphology and also the interfacial compatibility can be interpreted as follows. On one hand, according to the Guo-Hua Hu’s investigation [37], the morphology of blend of two immiscible polymers is determined by pellets melting/plasticizing speed, speed for deforming/breaking the polymer melt into small particles, and stabilizing speed. The above 3 factors follow a relationship of the first factor ≤ the second factor ≤ the third factor. Under the same processing conditions, all micropart samples have the similar pellets melting/plasticizing speed and the one with the smaller cavity size possesses the higher stabilizing speed due to the higher temperature gradient (faster cooling rate). Furthermore, the micropart with smaller cavity size has the higher dispersing speed than the one with bigger cavity size. This is because as the cavity size decreases, there is the significantly increasing shear rate, thus leading to much stronger dispersing effect on polymer melt droplets. As a result, when the mold cavity size decreases, the corresponding micropart has the much smaller PCL-dispersed phase domain size, thus resulting in better interfacial compatibility; on the other hand, the PCL-dispersed phase particles of the micropart with smaller cavity size are more easily stretched into microfibrils due to existence of the stronger shear stress field. The reason for this is that the shear force field of micropart with the smaller cavity size is much stronger (as shown in Figure 2, the shear rate of μ-PLA/PCL-15 micropart is nearly 4 times that of μ-PLA/PCL-26 micropart). Accordingly, under effect of the remarkably enhanced shear stress field, the low viscosity PCL-dispersed phase is much more easily stretched into microfibrils because of the interfacial tension (the PCL viscosity at the processing temperature is significantly less than that of PLA, which means a big viscosity ratio).

### 4.4. 2D-WAXD Analysis

**Figure 6:**
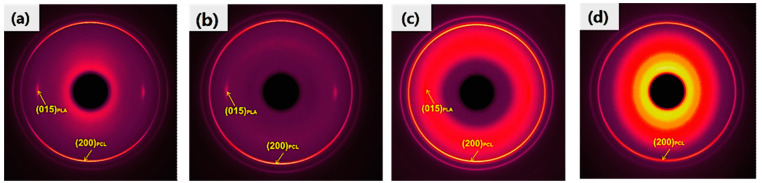
2D-WAXD patterns of the 80/20 micropart of (**a**) μ-PLA/PCL-15, (**b**) μ-PLA/PCL-20, and (**c**) μ-PLA/PCL-26, and (**d**) the shear layer of 80/20 macropart C-PLA/PCL. The flow direction is vertical.

**Figure 7:**
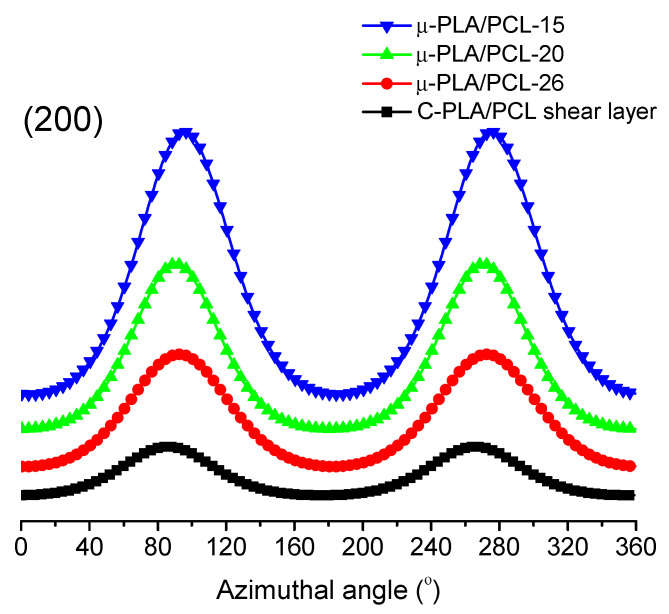
The (200) azimuthal intensity profiles of dispersed phase polycaprolactone (PCL) in microparts with different cavity size and shear layer of macropart.

Figure 6 shows the 2D-WAXD patterns of PLA/PCL 80/20 microparts with different cavity size and the shear layer of macropart. It can be seen that there are almost two reflection patterns (rings or arcs) occurring in the 2D diffraction pattern of each sample, which represent the (015) crystal plane of PLA in the equatorial direction (inner pattern) and the (200) crystal plane of PCL meridian direction (outer pattern), respectively. The intensity of the reflection pattern of PLA (015) plane is much weaker than that of the dispersed phase PCL (200) plane, revealing that the orientation of matrix PLA macromolecular chains is also much inferior to that of PCL macromolecular chains. In addition, the shear layer of the conventionally injection-molded sample shows a full and dim Debye ring of PCL (200) plane, and the reflection pattern of the PLA (015) crystal plane even disappears, indicating the molecular orientation of the polymers PLA and PCL in the conventional macropart sample is not strong. Comparatively, the microparts with different cavity size show the clear reflection rings or arcs of PCL (200) and PLA (015) crystal plane. This indicates that the molecular orientation of polymers PLA and PCL in the microparts is stronger than that in the macropart, which can well explain why the DSC results show the much higher crystallinity of micropart than that of macropart. This is because the orientation of polymer macromolecular chains contributes to their crystallization. For the micropart with different cavity size, with decrease in cavity size (μ-PLA/PCL-26 → μ-PLA/PCL-15), the corresponding reflection pattern of the matrix PLA and particularly the dispersed phase PCL is changed from Debye ring to arc. The smaller cavity size would lead to the shorter and stronger reflection arc. Above results show that with decrease in cavity size, there are more numbers of PCL macromolecular chains orientated along the melt flow direction and the degree of orientation of polymer macromolecular chains is also higher. The reason for this is that, as the cavity size of micropart decreases, the specific surface area of the mold cavity would increase, resulting in more polymer melts interacting with the mold cavity. This, no doubt, would remarkably intensify the apparent melt shear rate and hence the shear stress field occurring in mold cavity (as shown in Figure 2), thus making more polymer macromolecular chains orientated along the melt flow direction and further the polymer melt drops more efficiently extended. As a result, the degree of orientation would increase with decreasing the cavity size. The above results and analyses are verified by the azimuthal distribution of the intensity for PCL (200) crystal plane derived from the diffraction patterns of Figure 6, which is shown in Figure 7. The azimuthal peak intensity of PCL (200) crystal plane remarkably increases as the cavity size decreases, demonstrating the increasing degree of orientation of PCL macromolecular chains with decrease in cavity size.

In order to quantitatively determine the sample orientation parameter *f*, the function of Herman’s orientation [38] was applied here:

(6) $$f=\frac{3\langle \cos^{2}\varphi \rangle -1}{2}$$

where the orientation factor $\langle \cos^{2}\varphi \rangle$ is defined as below:

(7) $$\langle \cos^{2}\varphi \rangle =\frac{\int_{0}^{\pi /2}I\left(\varphi \right)\sin\varphi cos^{2}\varphi d\varphi }{\int_{0}^{\pi /2}I\left(\varphi \right)\sin\varphi d\varphi }$$

(8) cos φ=cosθ×cosμ

where θ is the Bragg angle, μ is the angle between the normal direction and the reference direction of crystal plane, and I(φ) is the reflection intensity at the azimuthal angle φ. When *f* = *1*, the macromolecular chains of polymers are orientated completely along the flow direction; when *f* = 0, the macromolecular chains of polymers are distributed randomly. Here, the PCL (200) crystal plane was used to calculate the orientation parameter *f*. The calculation results are given in Table 4. The orientation parameter *f* of PCL (200) crystal plane constantly increases with cavity size reducing, again proving that the decrease in cavity size is advantageous to the orientation of polymer macromolecular chains in micropart.

### 4.5. DMA Measurement

Dynamic mechanical analysis (DMA) method is an effective measurement to investigate the dynamic mechanical properties of the viscoelastic polymer material. The dynamic modulus and loss factor are closely related to the macroscopic properties of one material, including the stiffness and heat resistance, and also the changes in the microstructure and the macromolecular chains movement. From DMA, we can also know the information about structure and properties of polymers, such as damping characteristics, phase structure, phase transformation, and molecular relaxation.

**Figure 8:**
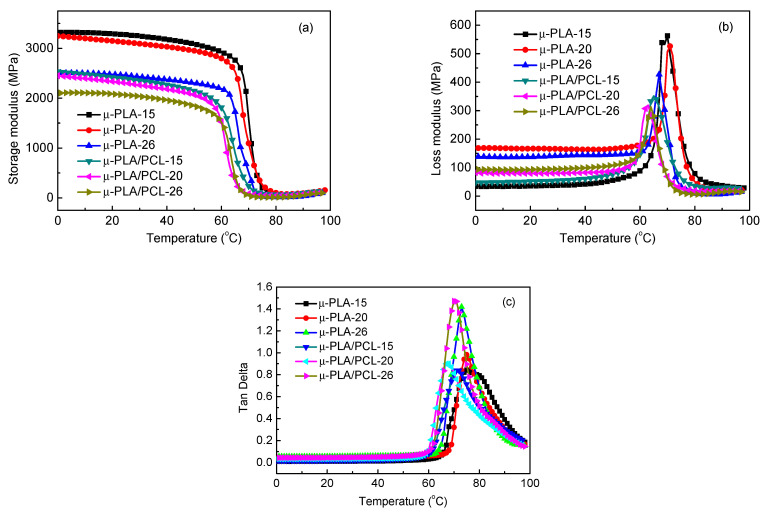
The dynamic mechanical analysis results of microparts with different cavity size as a function of temperature: (**a**) storage modulus, (**b**) loss modulus, and (**c**) tan δ.

Figure 8 shows the DMA curves of microparts of pure PLA and PLA/PCL blends with different cavity size as a function of temperature. The corresponding DMA parameters of different samples are summarized in Table 5. From Figure 8a, it is seen that the storage modulus of sample decreases with temperature increasing, showing anti-S dependence. The storage modulus and loss modulus of μ-PLA are higher than those of μ-PLA/PCL due to the flexibility of PCL. It is further noted that the storage modulus and the peak loss modulus increase as the cavity size decreases for both μ-PLA and μ-PLA/PCL blend samples. This is because as discussed before, with decrease in cavity size, there are the stronger shearing force occurring in mold cavity and the resultant cooling rate is also significantly increased. This would compel more polymer macromolecular chains to be orientated along the melt flow direction. These orientated macromolecular chains are then instantly frozen due to not enough time for relaxing. The smaller the cavity size, the greater the orientation degree, and so does the crystallinity. As a result, when suffered from the external force, the resistance to the movement of chain segments is therefore increased, resulting in the increase in storage modulus and loss modulus. In the meantime, this also indicates that with decrease in cavity size, the rigidity of the micropart sample increases.

Before the glass transition, the sample is under a glass state and the mechanical loss factor (tan δ) of both pure PLA and PLA/PCL blend microparts does not change significantly. After the glass transition, the tan δ peak value of all pure PLA microparts sharply increases, indicating the remarkable increase in the mechanical loss (damping). As we know, the tanδ peak temperature is corresponding to the glass transition temperature (T_g_). From Figure 8 and Table 5, the T_g_ of μ-PLA-26 (72.9 °C) is lower than that of both μ-PLA-15 (75.3 °C) and μ-PLA-20 (75.0 °C). The reason for above changes is that, on one hand, the T_g_ reflects the transition of the polymer chain segments in the amorphous region from freeze to free movement; on the other hand, as similarly stated before, the smaller cavity size would lead to the higher crystallinity and orientation degree, which correspondingly confines the movement of the polymer molecular chains. Under effect of the external forces, the resistance to the movement of chain segments increases, thus increasing the mechanical loss and also the T_g_.

The microparts of PLA/PCL blend system show a similar changing tendency. The storage modulus and loss modulus of micropart (particularly for the former) increase with decrease in the cavity size. This reveals that under the effect of the external stress, the capability for the micropart to resist the deformation becomes stronger and the stiffness is enhanced. According to Figure 8 and Table 5, the T_g_ of μ-PLA/PCL-26, μ-PLA/PCL-20 and μ-PLA/PCL-15 is 70.5 °C, 71.3 °C, and 71.5 °C, respectively, showing a slightly increasing tendency (not obvious). However, the tanδ peak value decreases with decrease in cavity size, indicating that the damping property of PLA/PCL blend micropart decreases with cavity size. In the meantime, the addition of the flexible PCL reduces the interactions between the PLA macromolecular chains, enhancing the movement capability of the chain segments. As a result, the change of T_g_ is not significant.

Comparing the μ-PLA and μ-PLA/PCL blend micropart, it can be known that the storage modulus of μ-PLA micropart is higher than that of μ-PLA/PCL micropart, indicating that the addition of the flexible PCL decreases the stiffness of PLA/PCL blend micropart. In addition, the T_g_ of μ-PLA micropart is also greater than that of μ-PLA/PCL blend micropart, but the tanδ peak value of the former is less than that of the latter. This indicates that the addition of PCL reduces the rigidity and heat resistance of PLA micropart, but enhances the damping property of material. This is because the addition of the flexible PCL polymer weakens the interactions between PLA macromolecular chains, making the PLA macromolecular chains move more freely.

### 4.6. Mechanical Property

**Figure 9:**
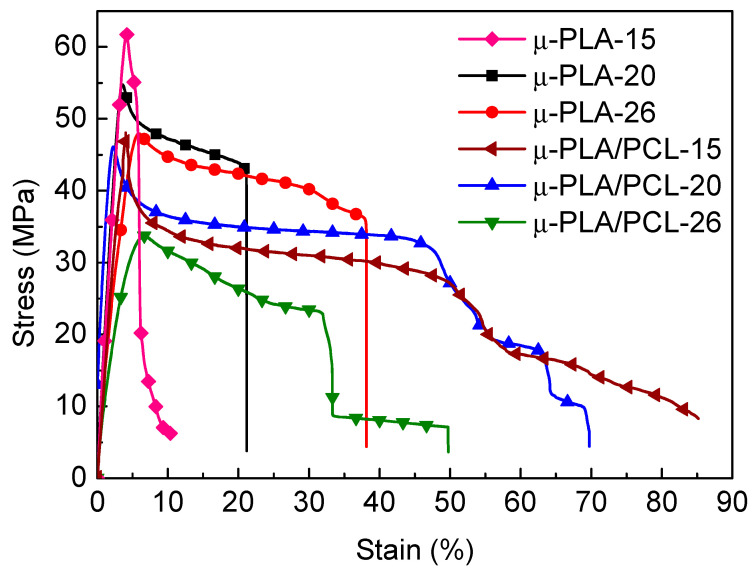
The stress-strain curves of microparts with different cavity size for pure PLA and PLA/PCL blends.

Figure 9 and Table 6 show the tensile properties and stress-strain curves of microinjection-molded microparts with different cavity size. It can be seen that, for pure PLA micropart, the tensile strength and Young’s modulus of μ-PLA-15 and μ-PLA-20 (smaller size) are obviously higher than those of μ-PLA-26 (bigger size), respectively. The increase degree for the tensile strength and Young’s modulus of μ-PLA-15 achieves 30.4% and 26.3%, respectively. However, the elongation at break of μ-PLA-15 and μ-PLA-20 is obviously lower than that of μ-PLA-26 (decreasing by 72.6% for the former). Generally speaking, the enhancement of the crystallinity and orientation degree of part would equally increase tensile strength and Young’s modulus, but would also equally lead to the decrease in the elongation at break and fracture toughness (we will evaluate the impact property of the related materials by using the impact tests later). As we know, during microinjection molding process, there are very strong shear force field generated in the mold cavity, which would promote the polymer macromolecular chains in melt state to be orientated along flow direction. Meanwhile, this orientation would also promote the formation of crystals and their growth. As a result, with decreasing the cavity size, the suffering shear force is enhanced, leading to the increase in the crystallinity and orientation degree. This is also the reason why the tensile strength and Young’s modulus of pure PLA micropart increase but its toughness decreases with the cavity size decreasing.

However, for PLA/PCL blend micropart, things are not completely identical. The tensile strength, Young’s modulus, and elongation at break of μ-PLA/PCL-15 and μ-PLA/PCL-20 are all greater than those of μ-PLA/PCL-26 (the former two microparts have the similar mechanical properties except for elongation at break), where the last one for μ-PLA/PCL-15 increases by 71.2%. On one hand, according to the results of Figure 2, the shear force field of the micro-mold cavity is strengthened with decrease in cavity size. The resultant crystallinity and orientation degree of the matrix PLA and the dispersed phase PCL in PLA/PCL blend micropart increase (Table 3 and Figure 7). As a result, the tensile strength and Young’s modulus of PLA/PCL micropart increase with decrease in the cavity size; on the other hand, the decrease in cavity size would also enhance the shear rate (shear force field) and the cooling rate occurring in the mold cavity. This would promote the dispersed phase PCL more homogeneously dispersed in PLA matrix, and simultaneously help the dispersed phase PCL form the fiber structures directionally aligning along flow direction. Under the effect of the external force, the formed fiber structures in PLA/PCL micropart would play a role in transferring and dissipating the stress. As a result, the elongation at break of PLA/PCL blend micropart increases with decrease in the cavity size, exhibiting a different changing tendency from the pure PLA micropart.

**Figure 10:**
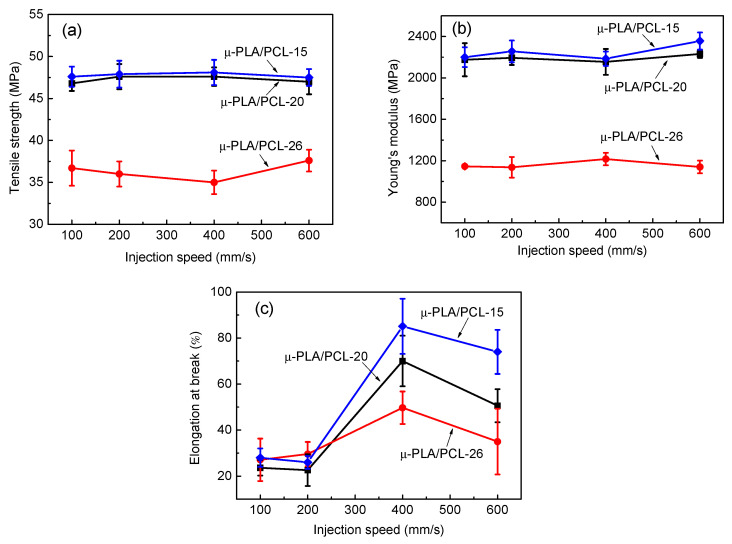
The mechanical properties of PLA/PCL blend microparts with different cavity size at different injection speed: tensile strength (**a**), Young’s modulus (**b**), and elongation at break (**c**).

**Figure 11:**
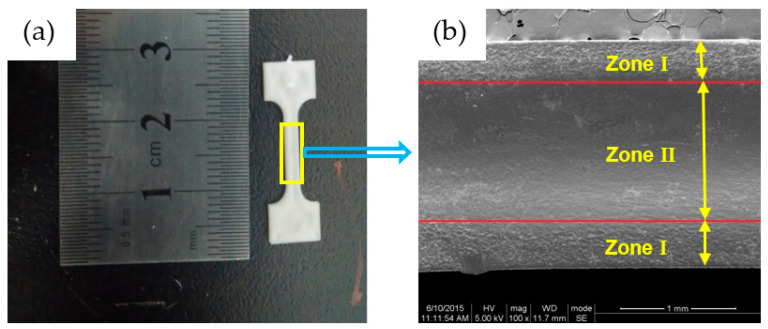
The digital photo (**a**) and SEM micrograph (**b**) of μ-PLA/PCL-26 micropart.

Figure 10 shows the influence of injection speed on the mechanical properties of PLA/PCL blend microparts with different dimension. As can be seen, totally, the μ-PLA/PCL-15 and μ-PLA/PCL-20 shows the significantly higher tensile strength and Young’s modulus than the μ-PLA/PCL-26 at various injection speed and the injection speed has a small influence on the corresponding mechanical performance. However, for the elongation at break, the injection speed has a small influence on μ-PLA/PCL-26 sample, but a relatively much bigger influence on μ-PLA/PCL-15 and μ-PLA/PCL-20 sample. With increasing injection speed, the change in the elongation at break of μ-PLA/PCL-26 is relatively smaller, and however, the one of μ-PLA/PCL-15 and μ-PLA/PCL-20 exhibits a significantly increasing tendency at higher than injection speed of 200 mm/s. The reason for this may be related to such a fact that in the smaller cavity size (μ-PLA/PCL-15 and μ-PLA/PCL-20), there are more numbers of PCL fiber structures formed in PLA matrix at higher than 200 mm/s due to the remarkably enhanced shear force field in the smaller mold cavity (Figure 5). For the higher mechanical performance of μ-PLA/PCL-15 and μ-PLA/PCL-20 than that of μ-PLA/PCL-26, the further study reveals that it is also possibly related to the warpage and surface shrinkage of the prepared μ-PLA/PCL-26 micropart, which is shown in Figure 11.

## 5. Conclusions

In order to investigate the influence of mold cavity dimension on the structure and properties of micropart under microinjection molding conditions, the micro-mold tools for tensile test with three cavity sizes were fabricated accordingly. The microinjection molding processing was successfully carried out on the PLA/PCL blend by using above three cavity-sizes of micro-mold tools. The micro-cavity shear rate, phase morphology, crystallization melting behavior and static/dynamic mechanical properties of PLA/PCL blend microparts with different dimension were deeply comparatively investigated. The results show that with decrease of the micro-mold cavity size from length 26 mm to 15 mm, the corresponding shear rate and temperature gradient increase significantly, indicating the remarkable enhancement in the shear stress field and the melt solidification rate in mold cavity. The SEM morphology and 2D-WAXD results show that the decrease in the cavity dimension is beneficial for fibrillation of the PCL-dispersed phase, improvement of interfacial compatibility and in situ formation of more numbers of PCL fiber-like structures with higher orientation degree. The above structure changes can be well correlated with the enhanced shear force field and temperature gradient due to the reduction in cavity size, as mentioned before, and would result in much better mechanical properties. DSC measurements show that the PLA crystallinity of blend micropart is much higher that of blend macropart. With decreasing the micro-mold cavity size, the PLA cold crystallization temperature significantly decreases and the PLA crystallinity increases, revealing the improvement of PLA crystallization performance with cavity size decreasing. In DMA test, as the cavity size decreases, the storage modulus and the loss modulus increase, indicating the increasing stiffness; the T_g_ of PLA also exhibits a slightly increasing tendency, revealing that the microinjection molding process could improve the heat resistance of micropart to a certain degree. The mechanical property measurements prove that the reduction of micro-mold cavity size remarkably enhances the mechanical performance of PLA/PCL blend micropart due to formation of more numbers of highly oriented fiber structures. As a result, it could be expected to realize the high performance of micropart through optimizing the micro-mold cavity size and structure.

## Figures and Tables

**Table 1:** The geometric parameters a^*^, b^*^, and f^*^ of rectangular die model for calculating shear rate [35].

h/w	Geometric Constants		
	a^*^	b^*^	f^*^
0.30	0.2991	0.7954	0.7297
0.35	0.2809	0.7750	0.7040
0.40	0.2659	0.7571	0.6820
0.45	0.2538	0.7414	0.6634
0.50	0.2439	0.7278	0.6478
0.55	0.2360	0.7163	0.6348
0.60	0.2297	0.7065	0.6242

Note: h/w is the ratio of height (h) to width (w) of the cross-section for the rectangular flow channel, and a^*^, b^*^, and f^*^ are the geometric constants parameters for rectangular die model.

**Table 2:** The cooling parameters of microparts with different cavity size.

Scheme	T_mold_ (s)	R (K/s)
μ-PLA/PCL-15	0.2	700
μ-PLA/PCL-20	0.6	233
μ-PLA/PCL-26	2.4	58.3

Note: T_mold_ is the time to reach the temperature of cavity wall and R is the cooling rate.

**Table 3:** The DSC parameters of microparts with different cavity size and macropart.

Scheme	T_cc_ (°C) PLA	ΔH_cc_ (J/g) PLA	T_m_		ΔH_m_ (J/g)		X_c,PLA_	X_c,PCL_
			PLA	PCL	PLA	PCL		
μ-PLA/PCL-15	80.4	9.87	168.0	61.5	35.65	19.30	34.7	69.3
μ-PLA/PCL-20	80.7	11.60	167.7	61.2	35.94	17.66	32.7	63.4
μ-PLA/PCL-26	82.1	12.34	168.0	61.3	34.21	17.40	29.4	62.5
C-PLA/PCL	86.8	17.47	165.1	56.9	29.47	12.36	16.1	44.4

Note: T_cc_, ΔH_cc_, T_m_ and ΔH_m_ are the cold crystallization temperature, cold crystallization melting enthalpy, melting temperature, and melting enthalpy, respectively.

**Table 4:** The orientation parameter *f* of microparts with different cavity size and shear layer of macropart.

Scheme	*f* of PCL (200) Plane
μ-PLA/PCL-15	0.398
μ-PLA/PCL-20	0.358
μ-PLA/PCL-26	0.279
C-PLA/PCL shear layer	0.191

**Table 5:** The glass transition temperature (T_g_) of pure polylactic acid (PLA) and PLA/PCL blend micropart with different cavity size in DMA test.

Scheme	T_g_ (°C)
μ-PLA-15	75.3
μ-PLA-20	75.0
μ-PLA-26	72.9
μ-PLA/PCL-15	71.5
μ-PLA/PCL-20	71.3
μ-PLA/PCL-26	70.5

**Table 6:** The tensile properties of microparts with different cavity size for pure PLA and PLA/PCL blends.

Scheme	Tensile Strength (MPa)	Elongation at Break (%)	Young’s Modulus (MPa)
μ-PLA-15	61.7 ± 1.3	10.5 ± 5.1	2016 ± 132
μ-PLA-20	55.9 ± 1.6	26.7 ± 4	2026 ± 110
μ-PLA-26	47.3 ± 1.0	38.3 ± 6.1	1596 ± 122
μ-PLA/PCL-15	48.1 ± 1.5	85.1 ± 12	2186 ± 69
μ-PLA/PCL-20	47.6 ± 1.1	70.0 ± 11	2155 ± 125
μ-PLA/PCL-26	35.0 ± 1.4	49.7 ± 21	1216 ± 60

## References

[1] Piotter V., Mueller K., Plewa K., Ruprecht R., Hausselt J. (2002). Performance and simulation of thermoplastic micro injection molding. Microsyst. Technol..

[2] Piotter V., Holstein N., Plewa K., Ruprecht R., Hausselt J. (2004). Replication of micro components by different variants of injection molding. Microsyst. Technol..

[3] Yang C., Yin X.-H., Cheng G.-M. (2013). Microinjection molding of microsystem components: New aspects in improving performance. J. Micromech. Microeng..

[4] Giboz J., Copponnex T., Mélé P. (2007). Microinjection molding of thermoplastic polymers: A review. J. Micromech. Microeng..

[5] Heckele M., Schomburg W.K. (2003). Review on micro molding of thermoplastic polymers. J. Micromech. Microeng..

[6] Yeh J.-T., Wu C.-J., Tsou C.-H., Chai W.-L., Chow J.-D., Huang C.-Y., Chen K.-N., Wu C.-S. (2009). Study on the Crystallization, miscibility, morphology, properties of poly (lactic acid)/Poly (ε-caprolactone) blends. Polym. Technol. Eng..

[7] Cai J., Liu M., Wang L., Yao K., Li S., Xiong H. (2011). Isothermal crystallization kinetics of thermoplastic starch/poly (lactic acid) composites. Carbohydr. Polym..

[8] Haroosh H.J., Chaudhary D.S., Dong Y. (2011). Electrospun PLA/PCL fibers with tubular nanoclay: Morphological and structural analysis. J. Appl. Polym. Sci..

[9] Al-Mulla E.A.J., Ibrahim N.A.B., Shameli K., Bin Ahmad M., Zin Wan Yunus W.M. (2014). Effect of epoxidized palm oil on the mechanical and morphological properties of a PLA–PCL blend. Res. Chem. Intermed..

[10] Xu H., Zhong G.-J., Fu Q., Lei J., Jiang W., Hsiao B.S., Li Z.-M. (2012). Formation of shish-kebabs in injection-molded poly (l-lactic acid) by application of an intense flow field. ACS Appl. Mater. Interfaces.

[11] Chavalitpanya K., Phattanarudee S. (2013). Poly (Lactic Acid)/Polycaprolactone blends compatibilized with block copolymer. Energy Procedia.

[12] Chee W.K., Ibrahim N.A., Zainuddin N., Rahman M.F.A., Chieng B.W. (2013). Impact toughness and ductility enhancement of biodegradable poly (lactic acid)/Poly (?-caprolactone) blends via addition of glycidyl methacrylate. Adv. Mater. Sci. Eng..

[13] Noroozi N., Schafer L.L., Hatzikiriakos S.G. (2012). Thermorheological properties of poly (ε-caprolactone)/polylactide blends. Polym. Eng. Sci..

[14] Chen C.C., Chueh J.Y., Tseng H., Huang H.M., Lee S.Y. (2003). Preparation and characterization of biodegradable PLA polymeric blends. Biomaterials.

[15] Broz M.E. (2003). Structure and mechanical properties of poly (D,L-lactic acid)/poly (ε-caprolactone) blends. Biomaterials.

[16] Yang J.M., Chen H.L., You J.W., Hwang J.C. (1997). Miscibility and crystallization of poly (L-lactide) Poly (ethylene glycol) and Poly (L-lactide) Poly (ε-caprolactone) blends. Polym. J..

[17] Wang L., Ma W., Gross R.A., McCarthy S.P. (1998). Reactive compatibilization of biodegradable blends of poly (lactic acid) and poly (ε-caprolactone). Polym. Degrad. Stab..

[18] Semba T., Kitagawa K., Ishiaku U.S., Hamada H. (2006). The effect of crosslinking on the mechanical properties of polylactic acid/polycaprolactone blends. J. Appl. Polym. Sci..

[19] Ji D., Liu Z., Lan X., Wu F., Xie B., Yang M. (2013). Morphology, rheology, crystallization behavior, and mechanical properties of poly (lactic acid)/poly (butylene succinate)/dicumyl peroxide reactive blends. J. Appl. Polym. Sci..

[20] Imai S., Hirai Y., Nagao C., Sawamoto M., Terashima T. (2018). Programmed self-assembly systems of amphiphilic random copolymers into size-controlled and thermoresponsive micelles in water. Macromolecules.

[21] Jiang Z., Liu H., He H., Ribbe A.E., Thayumanavan S. (2020). Blended Assemblies of amphiphilic random and block copolymers for tunable encapsulation and release of hydrophobic guest molecules. Macromolecules.

[22] Savagian L.R., Österholm A.M., Shen D.E., Christiansen D.T., Kuepfert M., Reynolds J.R. (2018). Conjugated polymer blends for high contrast black-to-transmissive electrochromism. Adv. Opt. Mater..

[23] Ding W., Chen Y., Liu Z., Yang S. (2015). In situ nano-fibrillation of microinjection molded poly (lactic acid)/poly (ε-caprolactone) blends and comparison with conventional injection molding. RSC Adv..

[24] Liu F., Guo C., Wu X., Qian X., Liu H., Zhang J. (2011). Morphological comparison of isotactic polypropylene parts prepared by micro-injection molding and conventional injection molding. Polym. Adv. Technol..

[25] Liu Z., Chen Y., Ding W., Zhang C. (2015). Filling behavior, morphology evolution and crystallization behavior of microinjection molded poly (lactic acid)/hydroxyapatite nanocomposites. Compos. Part A Appl. Sci. Manuf..

[26] Chen S., Tsai R., Chien R., Lin T. (2005). Preliminary study of polymer melt rheological behavior flowing through micro-channels. Int. Commun. Heat Mass Transf..

[27] Xinchao Wang L., Qinxing Z., Changyu S., Qian L. (2013). Design and flow characteristic of micro injection molded tensile specime. Polym. Mater. Sci. Eng..

[28] Li S., Wang L., Li Q., Zhang Q., Shen C. (2013). Analysis of molding ability of the micro thin wall parts and some flow phenomena in filling stage of micro injection molding. Polym. Mater. Sci. Eng..

[29] Meister S., Drummer D. (2013). Influence of manufacturing conditions on measurement of mechanical material properties on thermoplastic micro tensile bars. Polym. Test..

[30] Shibata M., Inoue Y., Miyoshi M. (2006). Mechanical properties, morphology, and crystallization behavior of blends of poly(l-lactide) with poly (butylene succinate-co-l-lactate) and poly (butylene succinate). Polymers.

[31] Crescenzi V., Manzini G., Calzolari G., Borri C. (1972). Thermodynamics of fusion of poly-β-propiolactone and poly-ϵ-caprolactone. comparative analysis of the melting of aliphatic polylactone and polyester chains. Eur. Polym. J..

[32] Wu C.-H., Liang W.-J. (2005). Effects of geometry and injection-molding parameters on weld-line strength. Polym. Eng. Sci..

[33] Kuo H.-C., Jeng M.-C. (2010). Effects of part geometry and injection molding conditions on the tensile properties of ultra-high molecular weight polyethylene polymer. Mater. Des..

[34] Xu B., An X.Y., Li L.C., Li G.M. (2014). Viscous dissipation of polymer melt in micro channels and macro channels. Key Eng. Mater..

[35] Son Y. (2007). Determination of shear viscosity and shear rate from pressure drop and flow rate relationship in a rectangular channel. Polymers.

[36] Frick A., Stern C., Michler G., Henning S., Ruff M.J.M.S., Pascalult J.P. (2010). Study on flow induced nano structures in iPP with different molecular weight and resulting strength behavior. Macromolecular Symposia.

[37] Li H.X., Hu G.H. (2001). The early stage of the morphology development of immiscible polymer blends during melt blending: Compatibilized vs. uncompatibilized blends. J. Polym. Sci. Part B Polym. Phys..

[38] Picken S.J., Aerts J., Visser R., Northolt M.G. (1990). Structure and rheology of aramid solutions X-ray scattering measurements. Macromolecules.

